# Comparison of big funnel and individualized stents for management of stomach cancer with gastric outlet obstruction

**DOI:** 10.1097/MD.0000000000013194

**Published:** 2018-11-30

**Authors:** Ding Shi, Jianping Liu, Xujun Hu, Yongpan Liu, Feng Ji, Yinsu Bao, Daxin Guo

**Affiliations:** a Department of Gastroenterology, Ningbo No. 2 Hospital, Ningbo; b Department of Gastroenterology, The First People's Hospital of Yuhang District; c Department of Gastroenterology, The First Affiliated Hospital of Zhejiang University, Hangzhou; d Department of Gastroenterology, The First Affiliated Hospital of Henan College of Traditional Chinese Medicine, Zhengzhou, China.

**Keywords:** gastric outlet obstruction, stents, stomach cancer

## Abstract

**Background:**

Previous studies reported a similar rate of complications, including stent migration and obstruction, between individualized stents and the standard uncovered stents in gastric outlet obstruction (GOO) caused by distal stomach cancer. The objective of this study was to evaluate the efficacy and safety of funnel stents for management of GOO caused by distal stomach cancer.

**Methods:**

This study was designed as a multicenter, controlled, prospective, and randomized clinical trial involving 4 hospitals. The individualized stent group (44 cases) received cup and funnel covered stents, and the funnel group (44 cases) received only funnel covered stents for management of GOO caused by distal gastric cancer.

**Results:**

All patients with GOO were treated with cup and funnel stents according to their assigned groups. The rate of GOO resolution was 100% in the funnel group and 97.7% in the individualized stent group. Stent obstruction caused by tumor ingrowth was observed in 1 patient in the individualized stent group, and proximal partial stent migration was observed in 1 patient in each group. Stent obstruction caused by tumor ingrowth was observed in 1 patient in the individualized stent group. There was no statistical difference in stent migration, obstruction, and survival between groups.

**Conclusion:**

Big funnel stents and individualized stents resulted in similar shaping effect and prevention of stent migration and obstruction, suggesting that funnel shaped stents can be used to treat cup or funnel shaped GOO caused by distal stomach cancer.

## Abstract 2

Supplemental Digital Content is available in the text

## 1 Introduction

In the last decade, stents have become one of the best treatments for malignant gastric outlet obstruction (GOO).^[1–5]^ A meta-analysis of 1281 patients showed that stent placement is a valid treatment option for the palliation of GOO, and a prospective study showed that stents improved quality of life for GOO patients.^[6]^ Other studies suggested that gastrojejunostomy should be considered as a treatment option for patients with a long life expectancy and could improve prognosis, but a short life expectancy or contraindication for surgery limits this option in many patients.^[7,8]^ Stent placement was associated with higher reintervention rates compared with surgery in some studies.^[9]^ However, the high migration rate of covered stents and the high tumor in growth rate of uncovered stents are not only major defects of standard stents, but are also the main factors leading to higher reintervention rates.^[10–16]^ Therefore, the reduction of stent migration and reobstruction rate is expected to improve stent efficacy in the treatment of malignant GOO.

Many researchers have improved metal stents for treatment of malignant GOO, but these improvements have not addressed the problems of stent migration and restenosis.^[17,18]^ In our previous studies, individualized stents were better than standard stents for preventing tumor ingrowth and stent migration, which reduced the reintervention rate.^[19,20]^ Individualized stents were designed according to the shapes of the proximal stomach cavity (“to measure”) so they could fit well in the remnant stomach cavity and provide a good pathway for the passage of food.^[19,20]^ We observed 2 basic shapes of the proximal cavity of GOO caused by distal stomach cancer in our studies: cup shaped and funnel shaped, and thus the stents for GOO treatment were designed as a cup or a funnel. Both stents demonstrated effectiveness in obstruction resolution.^[19,20]^ However, the design and production process of individualized stents is complicated. If individualized stents could be replaced by single shaped stents that have the same safety and clinical effects, then stent design and production would be simplified. Considering that design and production of funnel stents is relatively simpler, we hypothesized that funnel stents could be considered for all shapes of GOO in patients with gastric cancer. In the current study, we compared the efficacy and safety of funnel stents to individualized stents for treatment of non-resectable distal gastric cancer with GOO. In particular, we focused on shaping effect, technical success, clinical success, and adverse events rate.

## 2 Methods

This study was designed as a multicenter, controlled, prospective, and randomized clinical trial involving 4 large hospitals in China. All authors had access to the study data, reviewed, and approved the final manuscript.

### 2.1 Patients

This study was conducted between November 2014 and December 2016 and was approved by the Ethics Committees of the 4 involved hospitals. All included patients provided informed consent. Patient inclusion criteria were: gastric cancer, symptomatic GOO, obstruction located in the antrum and pylorus, and patients who were not candidates for surgery because of distant tumor metastasis or severe comorbidities. Patients were excluded if they: presented mild symptoms including liquid oral intake, had gastrointestinal perforation, another intestinal obstruction origin, or diabetes, or used pro-motility drugs, or had severe comorbidities that were contraindications for stenting procedure.

The patients were randomly divided into 2 groups: individualized stent group that received cup and funnel covered stents, and funnel stent group that received funnel covered stents. Yongpan Liu used block randomization with a block size of 6 to develop a random allocation sequence according to the previous randomization list. The random distribution sequences were placed in serially numbered, opaque, sealed envelopes and kept confidential. YL was only involved in generating random allocation sequences, and DG was only involved in patient allocation. The endoscopic physician identified if the subjects met the inclusion criteria and then informed DG, who sequentially opened the envelope and assigned the subjects to the corresponding groups. The operators were informed that the patient groups were already assigned and were not involved in patient group allocation. Doctors who were involved in patient follow-up were blinded; the operating doctors were un-blinded. Esophagogastroduodenoscopy was done to determine the size and shape of gastric obstructions, which were estimated and scored according to the Song et al^[21]^ system: a score of 0 denoted the ability to eat a normal diet; 1, the ability to eat solid food; 2, the ability to eat soft food; 3, the ability to swallow liquids only; 4, no oral intake without vomiting; 5, no oral intake with vomiting.

### 2.2 Stent design

**Figure 1:**
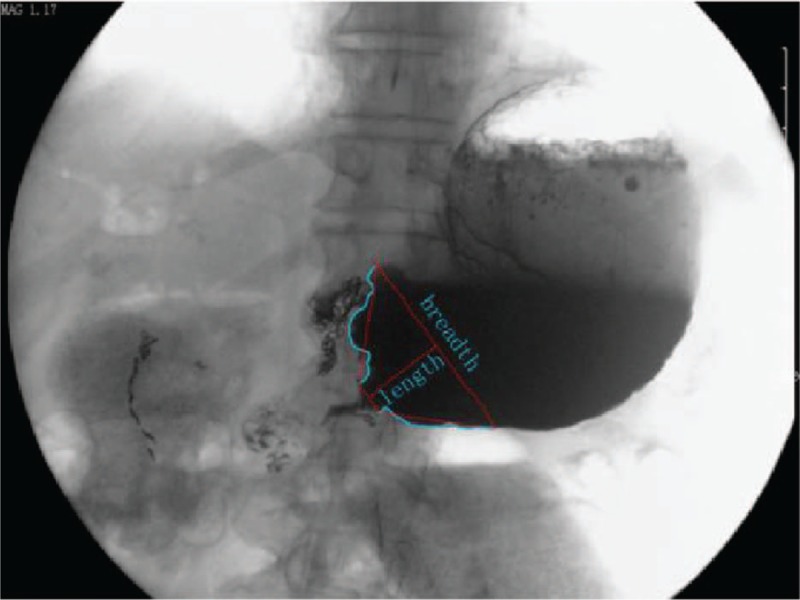
Examples of cup shapes in proximal lumens of GOO. The shape of the proximal lumen in the GOO could be regarded as funnel shape if the radian of the residual gastric cavity wall was ignored. GOO = gastric outlet obstruction.

Esophagogastroduodenoscopy was performed <3 days before stent design in order to determine the GOO shape. Esophagogastroduodenoscopy and stent design have been described in our previous studies.^[19,20]^ As determined by radiographic imaging, the maximum breadth of the obstruction was defined as the distance in the proximal residual gastric cavity, and the length was defined as the distance from the widest side of the residual gastric cavity to the sudden narrowing site. If the radian of the residual gastric cavity wall was ignored, the shape with wide proximal and narrow distal end could be regarded as a funnel shape (Fig. 1). Therefore, selection of funnel stents was the basic consideration in this study.

**Figure 2:**
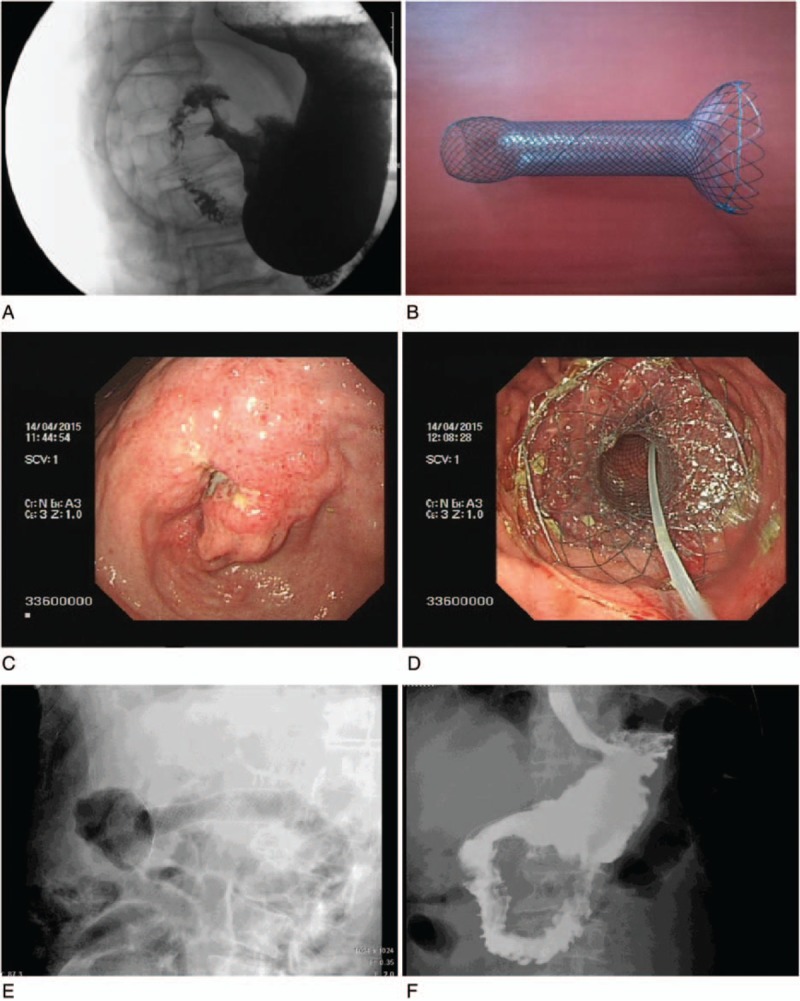
Use of a partially covered cup stent in treatment of cup shaped GOO. A, Distal gastric outlet cup shaped obstruction. B, Cup stent. C, Endoscopic view of a tumor in the distal gastric cavity. D, Endoscopic view of the proximal cup stent at the pyloric area. E, Confirmation of stent deployment by fluoroscopy. D, Barium contrast radiography shows contrast agent filling the stent. GOO = gastric outlet obstruction.

**Figure 3:**
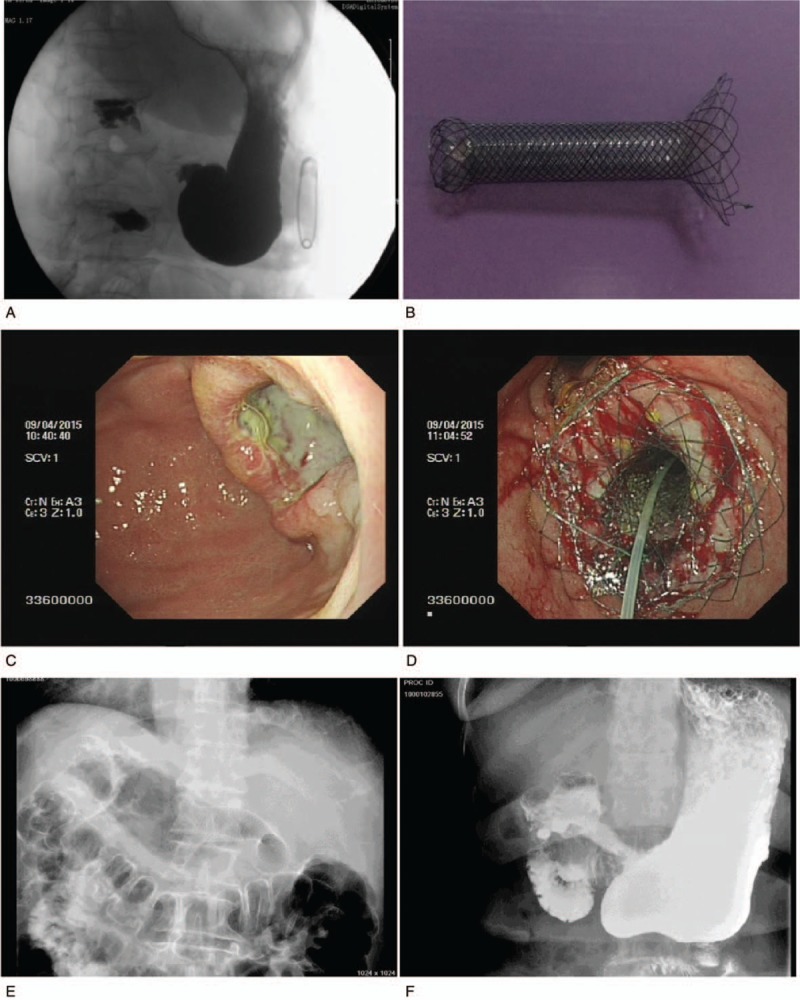
Use of a partially covered funnel stent in treatment of cup shaped GOO. A, Distal antrum cup-shaped obstruction. B, Model of funnel stent used to resolve the obstruction. C, Endoscopic view of a tumor in the gastric antrum. D, Endoscopic view of the proximal funnel stent at the pyloric area. E, Confirmation of stent deployment by fluoroscopy. D, Barium contrast radiography shows contrast agent filling the stent. GOO = gastric outlet obstruction.

**Figure 4:**
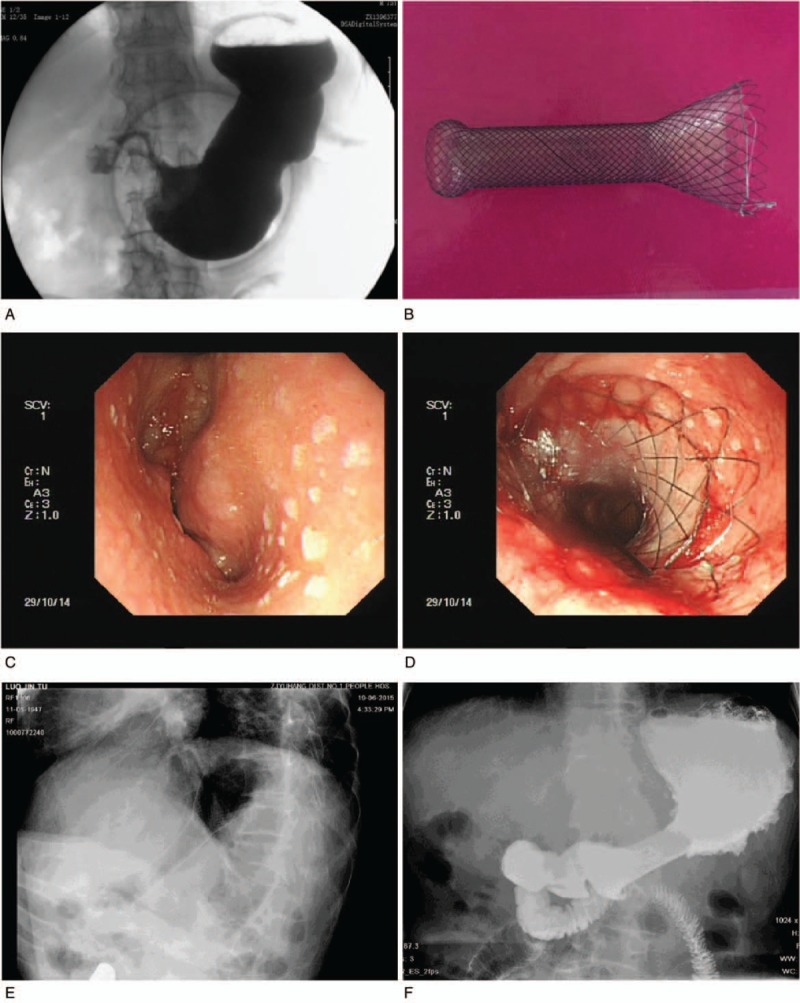
Use of a partially covered funnel stent in treatment of funnel shaped GOO. A, A 4 cm funnel shaped obstruction without contrast in duodenum. B, Model of funnel stent used to resolve the obstruction. C, Endoscopic view of the gastric tumor. D, Endoscopic view of the proximal funnel stent at the pyloric area. E, Confirmation of stent deployment by fluoroscopy. D, Barium contrast radiography shows contrast agent filling the stent. GOO = gastric outlet obstruction.

The standards for stent size design were 5 mm beyond the maximum breadth and length of the stomach cavity of the proximal cup and funnel of the obstruction, respectively. Thus, the stent diameter and length were equal to the maximum breadth and length of the obstruction cup or funnel plus 5 mm. Cup shaped (Fig. 2B) and funnel-shaped stents (Fig. 3B) (custom made, price $949 per stent, Micro-tech [Nanjing] Co., Ltd., Nanjing, Jiangsu, China) were used in the individualized stent group, according to the shapes of the proximal GOOs. All stents (custom made, price $949 per stent, Micro-tech [Nanjing] Co., Ltd., Nanjing, Jiangsu, China) in the funnel stent group were funnel-shaped (Fig. 4B). The distal portion of all stents was semispherical, with a length of 20 mm and a diameter of 28 mm. The body of the stent was 20 mm in diameter and 100 mm in overall length of the stent, with a polyethylene membrane covering. The stents were mounted on a delivery system with an outer diameter of 6 mm and an overall length of 130 to 180 cm.

### 2.3 Procedure

Esophagogastroduodenoscopy was conducted through a biliary guide wire, and then a super-stiff metal guide wire (MTN-Qf-90/42-b, Microtech [Nanjing] Co., Ltd, Nanjing, Jiangsu, China) was introduced and the endoscope removed to allow the delivery system (MTN-CR-6.0/180, Micro-tech [Nanjing] Co., Ltd., Nanjing, Jiangsu, China) to pass over the metal wire. The endoscope was inserted again to confirm placement. All stents were deployed under endoscopic and fluoroscopic guidance. It was important to maintain the position of the guide wire while the delivery system was inserted. If a stent insufficiently passed through the stricture, a second stent was inserted to complete the distance. After placement of all stents, endoscopy was performed to check stent proximal location (Figs. 2D, 3D, and 4D), and the stents were adjusted if the stent did not fit the proximal gastric lumen.^[19]^ Fluoroscopic views were obtained immediately after stent placement (Figs. 2E, 3E, and 4E).

### 2.4 Follow-up

Abdominal radiograph and esophagogastroduodenoscopy were performed 1 to 3 days after intervention to check stent expansion and location (Figs. 2F, 3F, and 4F). The follow-up and the evaluation of gastric outlet obstruction score system (GOOSS) improvement were performed at day 7 after stent placement. Monthly telephone calls were made to assess food intake until patients’ deaths. In some cases, follow-up data were obtained from the patient's family every month via an interview with a doctor who was in charge of the patient's follow-up. If nausea and vomiting were reported, the patient was inspected by endoscopy or radiography to confirm the presence of GOO recurrence and/or stent migration.

### 2.5 Outcome measurements

Stent dysfunction rate in GOO patients was used to determine primary outcomes. Secondary outcomes were based on clinical effectiveness as determined by the GOOSS: improvement in GOOSS ≥2 grades were considered effective, with coverage rate of the stent cup and funnel over the proximal lesion (endoscopy showed that the lesions in the residual antral cavity of GOO were completely covered by the stent cup or funnel), shaping effect (stent cup or funnel fit into the residual antral cavity; a barium study showed that the junction between the residual stomach cavity and the proximal end of the stent was smooth and regular), complications related to the procedure (immediate, major hemorrhage, perforation), technical success, and patient survival time.

### 2.6 Statistical analysis

Stent dysfunction rate in GOO patients after stent placement should be 27.3% according to the literature.^[22]^ In our previous studies, the stent dysfunction rate of individual stents in the treatment of malignant GOO was 4.2%.^[19,20]^ The minimum sample size of this study was 44 cases in each group. Statistical analyses were performed using SPSS for Windows (version 11.0. SPSS Inc., Chicago, IL). Continuous variables are expressed as means ± standard deviation (SD) and compared using the Student *t* test for 2 independent samples. Categorical data are expressed as n (the number of cases) or % and compared with the Chi square test or Fisher test. A *P* value <.05 was considered statistically significant.

## 3 Results

### 3.1 Patient characteristics

Of the 96 patients, 5 patients refused stent implantation and 3 patients were not suitable for stenting because of peritonitis or severe comorbidity. Eighty-eight patients were included in this study (44 cases in each group). Participant flow is shown in Supplementary Figure 1. Cup-shaped or approximate cup-shaped GOOs were observed in 34 patients in the individualized stent group (Fig. 2A and C) and in 33 patients in the funnel stent group (Fig. 3A and C), respectively. Funnel-shaped or approximate funnel-shaped GOOs were found in 10 patients in the individualized stent group and in 11 patients in the funnel stent group, respectively (Fig. 4A and C). The maximum breadth and length of the obstruction cup and funnel are shown in Table 1.

**Table 1:**
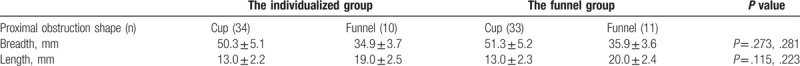
Number of cases and obstruction dimensions.

Table 2 shows that both groups were similar in terms of demographics, degree of differentiation, staging, chemotherapy use, and proximal gastric cavity shape of GOO (*P* > .05). In this study, 5 of the 88 included patients received chemotherapy after stent placement. There were no statistical differences before stent placement between the 2 groups. Thirty-four patients received cup-shaped stents and 10 patients received funnel-shaped stents in the individualized stent group. All patients in the funnel stent group received funnel-shaped stents. The whole stricture segment could be traversed by one stent in all patients in whom the stent could be inserted and no second stents were used.

**Table 2:**
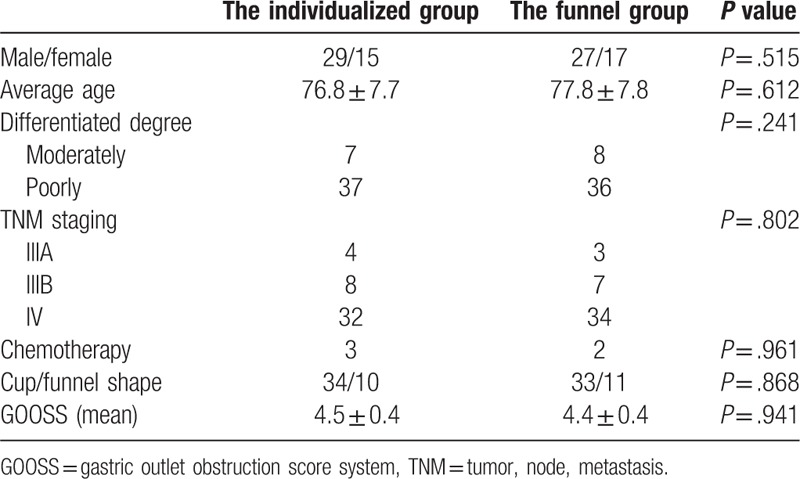
Patient characteristics.

### 3.2 Technical and clinical outcomes

The efficacy and complication rates of the 2 groups are shown in Table 3. Technical success was defined as adequate placement of the self-expandable metal stent across the stenosis, as confirmed by a combination of endoscopy and fluoroscopy. In each group, one stent could not be implanted successfully because the stent delivery system looped into the dilated gastric fundus. All stents were trans-pyloric. Full coverage was considered when the edge of the proximal lesion of the GOO was exceeded by the proximal edge of the stent cup or funnel (Figs. 2D, 3D, and 4D). The coverage rate was determined by the ratio of full covered lesions to the total number of patients in each group. The shaping effect was obtained in all cases except for 1 in the individualized stent group (Figs. 2D, 3D, and 4D).

**Table 3:**
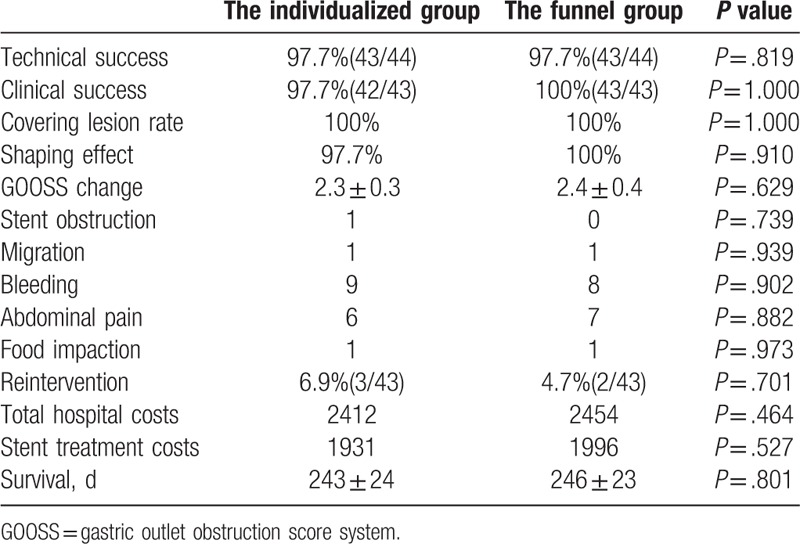
Efficacy and complications.

In 1 patient in the funnel group, a proximal funnel stent initially protruded into the wide gastric cavity because of inaccurate implantation, but the stent was adjusted (Supplementary Figure 2).

Clinical success was determined by resolution of obstructive symptoms and the ability to restart a low diet after stent placement. Only 1 patient in the individualized stent group did not have symptoms improvement. The other patients showed improvements of ≥2 grades in the level of dietary intake at day 7 after stent placement. There was no statistical difference in terms of symptoms improvement at day 14 after stent placement between the 2 groups (Table 3). In the control image studies, we confirmed adequate implantation and contrast medium passage through obstruction in both groups (Figs. 2F, 3F, and 4F).

### 3.3 Stent complications

Stent obstruction caused by tumor ingrowth was observed in 1 patient in the individualized stent group during the follow-up period. Tumor ingrowth was observed in the distal uncovered section of 1 cup shaped stent. In cases of restenosis, a standard uncovered stent was reinserted to overlap a primary stent. No patient presented stent obstruction by tumor overgrowth. Proximal partial stent migration appeared in 1 patient with a cup stent in the individualized stent group and 1 patient in the funnel stent group, respectively. These 2 patients received chemotherapy after stent placement. We did not observe distal stent migration. The time to develop proximal partial stent migration was 79 days in the individualized stent group and 113 days in the funnel group. There was no statistical difference in terms of stent migration and obstruction between the 2 groups. In the cases of stent migration, replacement stents were re-implanted after removal of the former ones.

### 3.4 Adverse events

Bleeding occurred in both groups, but there was no statistical difference. There were no serious adverse events such as perforation or major hemorrhage. Food impaction after stent placement was treated endoscopically.

### 3.5 Survival

The last patient in the study died in December 2016. Mortality (97.7%) was observed during follow-up with a survival time of 243 ± 24 days (30–399 d) in the individualized stent group and 246 ± 23 days (43–421 d) in the stent funnel group. There was 1 patient in each group who did not come to follow-up.

## 4 Discussion

The gastric cavity is different and remains wide even when occupied by gastric tumors. For this reason, stent migration and obstruction by tumor overgrowth is a common defect in standard stent ends at smaller diameters, and is necessary to increase stent proximal end diameters.^[21–27]^ In our previous studies, we designed individualized stents to treat GOO. Specifically, the goal was to prevent tumor ingrowth by covering the stent body with a membrane and prevent tumor overgrowth and distal stent migration by increasing the diameter at the proximal ends of the stents. Our studies demonstrated that individualized stents were superior or similar to the standard stents in preventing tumor ingrowth and stent migration.^[19,20]^ Moreover, the individualized stents had a superior effect on shaping the residual proximal gastric cavity, allowing gastric emptying.^[19,20]^ In this study, the results in the individualized stent group verified the above conclusions. However, the design and production of individualized stents is more complicated than standard stents, which is not conducive to clinical practice. Simplifying the design and production of stents is an unavoidable problem. In this study, the radian of the proximal residual gastric wall of GOO was ignored, which was regarded as a funnel shape with a wide proximal and a narrow distal end, which made measurement and judgment easier. Production of funnel stents is also simpler than cup stents given their regular shape. The present study demonstrated that funnel stents were similar to individualized stents with regards to clinical effect, preventing stent migration, stent obstruction, lesion coverage, and shaping effect. But, the funnel stent migration and reobstruction rate was significantly lower than other stents reported in previous studies.^[16,28–30]^ Moreover, the funnel stent is conducive to clinical practice. It has been previously reported that the cup stent had a higher proximal migration rate but not in our study, and the reason might be that our stents were specifically designed (larger distal ends with a diameter of 28 mm), which prevented stent proximal migration, while the distal ends of stents in the previous report were cylindrical.^[31]^ There were few cases of proximal partial stent migration in our study. This partial stent migration may be related to the longer covered membrane part of the stent and the shorter obstruction, or it might be related to chemotherapy. Moreover, 1 patient in the individualized stent group did not show improvements in symptoms after stent placement. The reason for failure of clinical success might be due to lack of propulsive peristalsis in a chronically obstructed stomach,^[32]^ or functional GOO due to neural involvement of the tumor.^[33]^

The survival time of patients is an important indicator of long-term effect of stent therapy. Compared with previous reports, the patients treated with funnel stent in this study have longer survival time.^[28,30,34,35]^ Many factors must be involved in the observed difference, for example, patients related factors. However, the main cause must be related with the funnel stents longer patency and less stent obstruction caused by tumor ingrowth or overgrowth.

Cost analysis is also an important consideration when selecting the appropriate stent. The median cost of treatment with funnel stents was $1996.00, which was comparable to individualized stents and a standard one in China.^[19,20]^ However, both endoscopic stenting and total hospital costs (including reinterventions) for funnel stents in this study are by far lower than other stents reported in other countries around the world.^[2,22,28,36–38]^ Therefore, funnel stents did not increase the total cost for patients with GOO.

In summary, funnel stents are equivalent to individualized stents, which have the advantages of preventing stent migration and reobstruction similar to the individualized ones. However, funnel stents are superior to other stents in terms of survival time, cost of stent treatment, and preventing stent migration or reobstruction. Moreover, while the shape of the residual stomach cavity is neglected, the design and manufacturing of funnel stents are more simplistic compared with the individualized stents. Therefore, funnel stents should be recommended for GOO caused by distal gastric cancer.

A limitation of this study was that the funnel stents could only be used in the obstruction of antral stricture, which could not be implanted by endoscopic channel (through-the-scope), and the covered membrane length of the stent had not been individualized. Furthermore, follow-up was conducted by monthly telephone calls, which may have underestimated the rate of stent dysfunction.

In conclusion, we found that funnel stents are similar to individualized stents for lesion covering, shaping effect, survival, and preventing stent migration and obstruction. These findings can be applied to treating cup or funnel shaped GOO caused by distal stomach cancer.

## Author contributions

**Data curation:** Xujun Hu, Yongpan Liu.

**Formal analysis:** Feng Ji, Yinsu Bao, Daxin Guo.

**Writing – original draft:** Jianping Liu.

**Writing – review & editing:** Ding Shi.

## Supplementary Material

Supplemental Digital Content
