# Supplementary material for: Comparison of big funnel and individualized stents for management of stomach cancer with gastric outlet obstruction
Source: Medicine (Baltimore). 2018 Nov 30;97(48):e13194. doi: 10.1097/MD.0000000000013194 (PMC6283228; doi:10.1097/MD.0000000000013194)
Supplement: Supplemental Digital Content [file medi-97-e13194-s001.pdf]

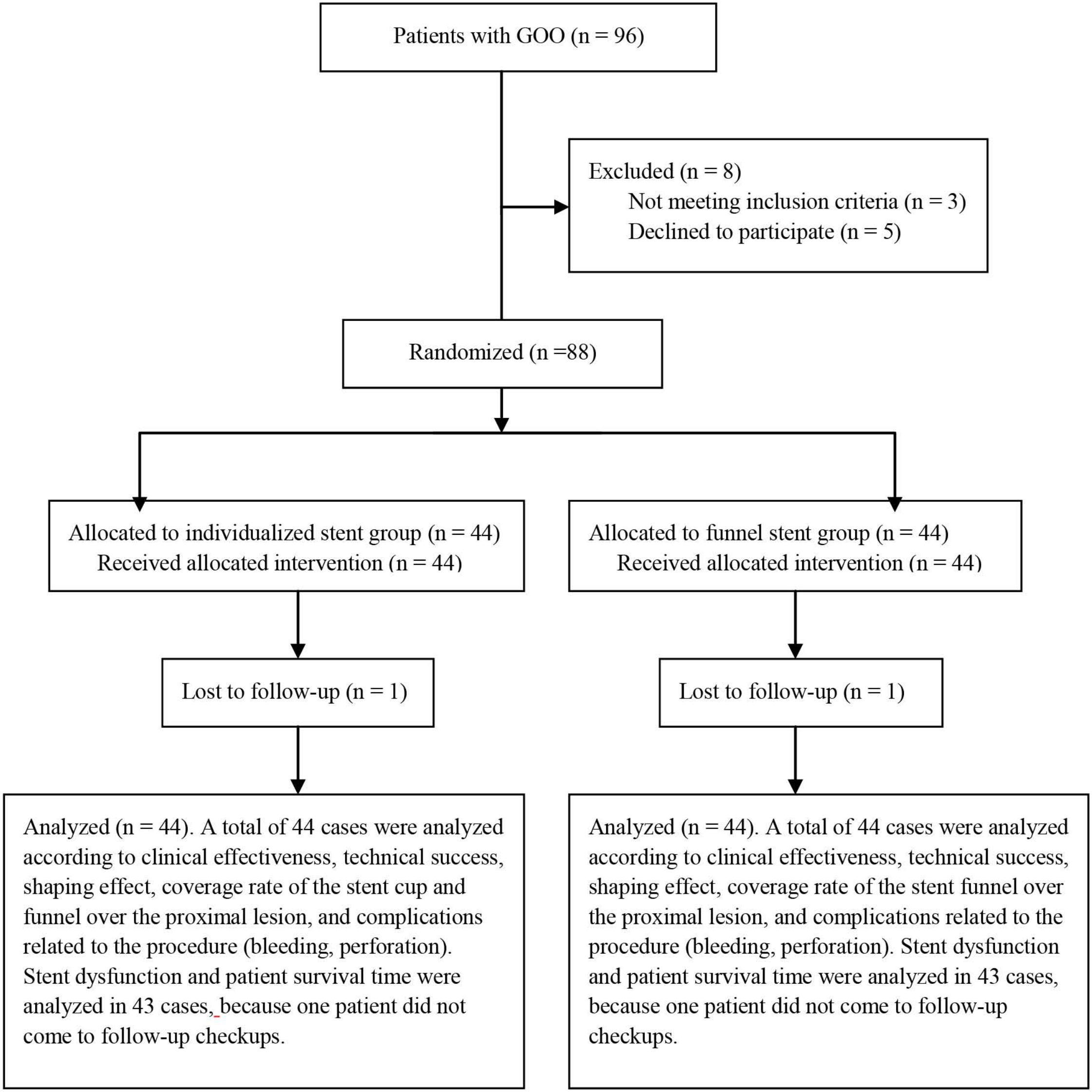
Patients with GOO (n = 96)
Excluded (n = 8)
Not meeting inclusion criteria (n = 3)
Declined to participate (n = 5)
Randomized (n =88)
Allocated to individualized stent group (n = 44)
Received allocated intervention (n = 44)
Allocated to funnel stent group (n = 44)
Received allocated intervention (n = 44)
Lost to follow-up (n = 1)
Lost to follow-up (n = 1)
Analyzed (n = 44). A total of 44 cases were analyzed according to clinical effectiveness, technical success, shaping effect, coverage rate of the stent cup and funnel over the proximal lesion, and complications related to the procedure (bleeding, perforation). Stent dysfunction and patient survival time were analyzed in 43 cases, because one patient did not come to follow-up checkups.
Analyzed (n = 44). A total of 44 cases were analyzed according to clinical effectiveness, technical success, shaping effect, coverage rate of the stent funnel over the proximal lesion, and complications related to the procedure (bleeding, perforation). Stent dysfunction and patient survival time were analyzed in 43 cases, because one patient did not come to follow-up checkups.

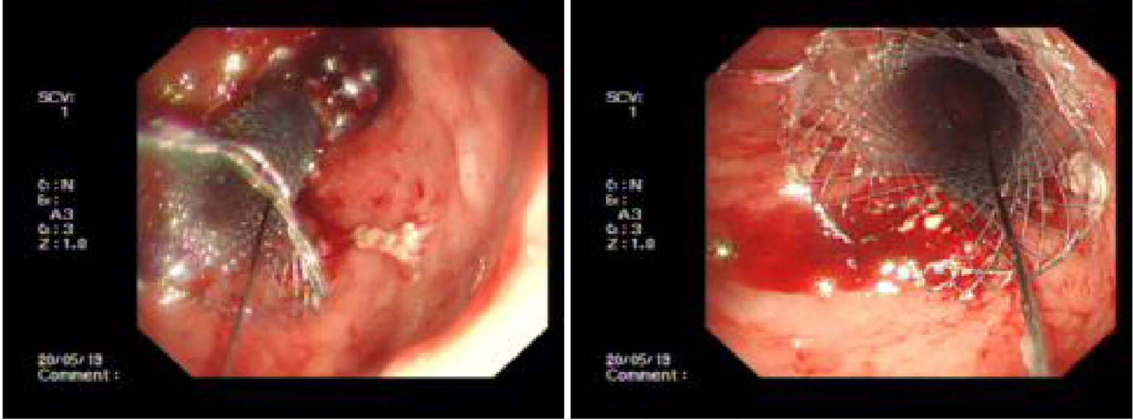

A B
